# School nurse preparedness for critical events – a multi-modal simulation training pilot

**DOI:** 10.1186/s41077-026-00432-z

**Published:** 2026-03-29

**Authors:** Samuel Gowan, Marcie Gawel, Barbara M Walsh

**Affiliations:** 1 Saint Louis University, Doisy College of Health Sciences, Physician Assistant Program, MO St Louis, U.S.; 2 Yale New Haven Hospital, Center for Injury and Violence Prevention, CT New Haven, U.S.; 3 Department of Pediatrics, Boston University Chobanian and Avedesian School of Medicine, Boston Medical Center, 801 Albany Street, 4th Floor, Boston, MA 02119 U.S.

**Keywords:** School emergency preparedness, Simulations for school nurses, In situ simulation, Simulated critical events in schools for nurses

## Abstract

School-based emergencies, notably school shootings, are emotionally taxing events for school nurses (SNs). As frontline healthcare providers, SNs must rapidly assess injuries, coordinate emergency response, support the school community, communicate with families and emergency medical services (EMS), and manage their emotions. Yet most SNs receive minimal standardized training to manage mass-casualty incidents effectively. In the United States, SNs in the Boston Public School System (BPSS), have experienced increasing penetrating trauma, prompting leadership to pursue targeted educational solutions. Although a U.S. prominence, school violence remains a global concern, emphasizing its worldwide relevance and underscoring the international need for school-based emergency preparedness. In response, an interprofessional team developed a tailored, integrated emergency curriculum informed by a collaborative needs assessment. Recognizing that medical crises occur more frequently than shootings, the curriculum intentionally addressed both traumatic and medical emergencies. The multimodal program prepared SNs to bridge care until EMS arrives and included two hours of interactive workshops followed by two realistic multi-victim simulations. Interprofessional faculty facilitated skills training, simulation, and debriefing while assessing adherence to evidence-based practices. Pilot findings revealed gaps in leadership, communication, triage, CPR/PALS initiation, and task delegation. Strong engagement led BPSS leadership to request continuation in 2024, with returning participants demonstrating improved command presence, delegation, and closed-loop communication. Next steps include formal program evaluation using validated assessment tools and expansion to incorporate disaster triage protocols and preparedness. Given global lack of standardized emergency training for SNs, this innovative curriculum offers a scalable, adaptable, impactful model for strengthening school-based emergency readiness worldwide.

**Supplementary Information:**

The online version contains supplementary material available at 10.1186/s41077-026-00432-z.

## Background

Everyday there are millions of children in the United States (US) and abroad attending school, and while on campus they are under the medical care of school nurses (SNs). It is important to note that while SNs have a broad scope of practice, and their clinical training adequately prepares them to care for children’s basic complaints, there is no uniformity in SNs requirements and certifications despite guidelines outlined by the National Association of School Nurses [1]. Moreover, during any on-campus medical incident, SNs are typically the primary responder until EMS arrives [2–4]. Two U.S. studies using similar methodologies (2005 and 2014) found that SNs felt less comfortable managing head injuries, seizures, cardiac arrest, and other emergent conditions [4–6]. Furthermore, there is inconsistent and unstandardized disaster management education in nursing curricula secondary to cost, lack of educators, and insufficient resources to sustain programming [6–8]. The literature demonstrates that SNs are not prepared to manage critical incidents in schools.

In addition to “typical” injuries at schools, an unfortunate new reality in the US is the increase of school/mass shootings and related deaths [9, 10]. While this issue is most significant in the US, there is clear evidence that this is a global phenomenon [11]. If a school shooting occurs on school grounds, it could escalate to a mass casualty event, referring to situations where the needs of those affected are larger than the available resources to care for the patients. SNs will manage the scene until EMS arrives, and those minutes prior to EMS support are vital to patient outcomes [12, 13]. Following analysis of the Sandy Hook Elementary School in 2012, an inter-professional (IP) team created the Hartford Consensus, a report illustrating strategies to improve victim survival [14, 15]. Subsequently, a Stop The Bleed© campaign ensued to train bystanders to intervene early as this improves survival rates for severe bleeding.

In 2022, the Boston Public School System (BPSS) identified this gap in training and sought help from local experts to address this problem. Leadership at BPSS contacted the Community Outreach Mobile Education Training program (COMET: www.cometsimulation.com) to create a tailored program for their staff given COMET’s expertise in pediatric emergency preparedness training [16, 17]. COMET embraced this opportunity as a vital curriculum innovation to facilitate SNs emergency response to a multi-victim event. We know that prepared trauma systems will have superior outcomes secondary to the investment in personnel education and emergency protocol development [18, 19]. Thus, investment in a program for the SNs to thrive in such an event is critical. The importance of this curriculum is also timely and relevant in the evolving culture of traumatic events at schools in both the US and global community. This gave the team an opening to consider a novel, flexible curriculum with potential reach beyond one school system given the geographic expanse of this problem. In this light, we hope to share the creation and implementation of a sample, educational innovative curriculum of multi-victim trauma education and response, with descriptive and observational outcomes that can be adapted, iterated and refined to any school site’s needs.

## Main body

### Curriculum development

An academic colleague connected BPSS with COMET. Collaborative discussions via intensive focus groups with nurse leadership from BPSS health department and the COMET team were undertaken to inform of school resources, concerns, traumatic incidents, school staffing, and prior educational offerings. Leaders within BPSS were interested in a comprehensive program that would allow SNs more facility approaching any traumatic school event using their limited resources pending EMS.

Following this needs assessment, an IP team (pediatric emergency medicine physicians and nurses, trauma specialists, child life, social work, and EMS), developed novel pilot training modules to educate SNs in clinical management of topics with which the SNs are less competent, using hands-on didactic and simulation methodologies. Simulation training was included because it provides a risk-free environment for SNs to practice life-saving measures and is proven to increase adherence to evidence-based practices in a real-life critical event [20, 21]. Creating mass casualty type events with simulation, allows SNs to immerse themselves in potential realistic scenarios, to practice skills, and identify knowledge gaps.

The curriculum was developed by an interdisciplinary team of pediatric trauma content experts. Realistic time constraints to move school nurses efficiently through the program informed the session design. Based on interdisciplinary consensus and school nursing leadership input, four workshop topics were identified as most relevant: splinting with what you have, Stop the Bleed©, neurologic emergencies, “the first 10 min” (seizure, syncope, concussion), and debriefing/de-escalation of critical events (Table 1). These 30-min rotating workshops used a multimodal format, combining discussion-based learning, guided practice, and hands-on skill application. Each workshop was facilitated by an interdisciplinary team aligned with its topic. The second part of the curriculum involved the development of two escalating, high-fidelity multi-victim simulations conducted in realistic, time-pressured environments. There was an array of acuity per simulation with medical cases interwoven. Boston EMS (BEMS) provided a standardized triage model for multi-victim events that was embedded into the training and reinforced during debriefs.

**Table 1:** Rotating Emergency-Related Workshops

Topic	Content	Faculty
A-Stop the bleed	Hands-on put on a tourniquet in a progressively chaotic scene, packing wounds	Staff: MDs, RNs, EMS
B-Seizure/Syncope/Concussion	Didactic- 10 min on each topic-1st 10 min at scene	Staff: MDs, RNs
C-Splint/Dressings	Hands-on splint-in progressively chaotic scene How to be creative with what you have	Staff: MDs, RNs, EMS
D-Debrief/De-escalation of critical events	Interactive discussion	Staff: Social Work, Child Life, Fire

Breaks down the four workshops. The first column is the title of the workshop. The second column describes what didactic content would be included in the workshop. The third column lists the specialists who supported the workshop

The curriculum design was grounded in Kolb’s Experiential Learning Theory and the INACSL Standards of Best Practice: Simulation Design, emphasizing progressive skill acquisition, application, and reflection. Debriefings followed the PEARLS framework (Promoting Excellence and Reflective Learning in Simulation) to enhance reflective learning, crisis management, and interdisciplinary communication as this was the standard approach of COMET educators [22]. This framework was intentionally selected to support SNs as nontraditional first responders, fostering clinical confidence, decision-making, and systems awareness in high-stress, resource-limited environments. The repeated program used the same format, but with additional victims to ensure all participants had direct, hands-on involvement. Table 2 outlines the initial simulation design elements across the scenarios.

**Table 2:** Simulation design elements across both scenarios

Elements	Description
Learning Objectives	1. Perform rapid primary assessment (ABCDE) and repeated reassessment across multiple victims with variable acuity 2. Prioritize care using mass-casualty and triage principles appropriate to school-based emergencies 3. Apply time-critical interventions, including hemorrhage control (direct pressure, tourniquet use, wound packing), splinting, seizure management, airway protection, and high-quality CPR 4. Demonstrate effective team leadership, delegation, resource utilization, and clear closed-loop communication under stress 5. Activate emergency response systems efficiently and prepare structured handoffs for EMS arrival
Scenario Fidelity	Environmental fidelity: School nurse office, hallway, classroom, and athletic field environments adapted with limited supplies to mirror real-world constraints; noise, movement, and distractors incorporated for realism Psychological fidelity: Hysterical teens, distressed peers, emotional intensity, uncertainty, time-pressure cues, and cognitive overload designed to simulate real-life stress Clinical fidelity: Mixed fidelity—live actors with moulage, task trainers for amputation and wound packing, medium- and high-fidelity manikins for VFib arrest; realistic bleeding, airway compromise, and seizure activity
Roles	Primary School Nurse (Team Leader): Directs triage, assigns roles, oversees interventions Secondary Nurse/Support Staff: Performs delegated skills (tourniquets, packing, splinting, CPR) Runner/Clerk/Coach/Teacher: Retrieves AED/bleeding kits, calls EMS, provides scene cues Victims: Mixed acuity (critical, delayed, minor); portrayed by student actors, staff, or manikins Faculty: RN, MD, Fire, Child Life, Social Work, EMS educators operating scenario flow, providing cues, and leading debrief
Assessment Focus	• Triage accuracy across multiple victims • Recognition of critical illness vs. non-critical distractors • Hemorrhage control technique and sequencing (pressure → packing → tourniquet) • Airway protection, seizure safety, and management of altered mental status • High-quality CPR, AED use, and coordination during cardiac arrest • Leadership presence, task delegation, and closed-loop communication • Frequent reassessment, vital-sign trending, and adjusting priorities based on changes
Debrief Method	Structured debrief using PEARLS and Advocacy–Inquiry frameworks with emphasis on psychological safety. Debrief focused on clinical reasoning, triage decisions, team communication, leadership, emotional responses, and resource-limited decision-making Facilitators linked participant actions to simulation-based learning principles (experiential learning, deliberate practice, fidelity alignment, mastery behaviors) and reinforced best practices for school-based emergency preparedness

### Pre-implementation work

The curriculum was planned on a weekend to ensure that SNs could participate without disrupting their weekday responsibilities and to avoid conducting an emotionally charged exercise while students were present. The selected date avoided major family or vacation conflicts to maximize attendance. A high school with ample classroom space for workshops and a large cafeteria for the simulations hosted the event.

Participation was open to all SNs, athletic trainers, and school administrators who would respond during a school emergency. Invitations were distributed through BPSS nursing leadership, and attendees selected one of two four-hour sessions held consecutively on the same day. Free contact hours were offered as an incentive, and all participants provided consent for video recording. Video review was planned for reflective analysis, focusing on session dynamics, skill application from the workshops, and team coordination during the scenarios. Post-session surveys were created to gather feedback assessing perceived impact and inform future curricular iterations.

The COMET team intentionally assembled a diverse interprofessional faculty reflective of the scenarios being taught. COMET partnered with BEMS, the first responders for the targeted schools, whose senior leadership helped recruit paramedics and EMTs to serve as facilitators and simulation participants. Additional volunteers included pediatric emergency nurses, fellows and physicians, emergency residents, medical students, social workers, child life specialists, and disaster medicine colleagues. Participation was completely voluntary. Faculty were assigned to workshops based on their topic expertise allowing multiple educators of diverse backgrounds. This team diversity was intentional, fostering broader perspectives and deepening experiential learning. The interprofessional faculty were equally divided between the two simulations. Recognizing that school-based mass casualty incidents (MCI) have shifted from rare to anticipated occurrences, this shared awareness galvanized the faculty to collaborate on a training initiative grounded in readiness, resilience, and community partnership.

### Implementation: workshops and simulation scenarios

The two-hour rotating workshops were structured around distinct learning objectives aligned with its focus area (Table 1). For example, the *Stop the Bleed©* session incorporated a brief didactic component followed by instruction on tourniquet selection and application, hands-on practice, and a final task execution in a simulated high-stress environment with realistic visual and auditory distractions (blood and noise).

The two rotating simulation events: 1) cafeteria school shooting, 2) school athletic event had medical emergencies woven in to broaden the experience with potential victims. These scenarios reinforced workshop objectives and prior training (including BLS/CPR), allowing school nurses to apply and integrate new and existing skills in realistic, high-stakes environments while managing team communication, prioritization, and patient care under pressure. Hysterical students were added as distractors though some had wounds that needed tending. The details of the victims (injury, actor vs mannikin) and expected actions are in Table 3.

**Table 3:** Triage categories, expected actions, and clinical prioritization across victims

*Victims: school shooting*	*Acuity/triage category*	*Expected actions by school nurse*
Teen with Panic/Anxiety Reaction Live actor	Minor / Low Priority	• Assign a layperson or staff member to remain with student • Provide reassurance and psychological support • Ensure scene safety and keep patient away from critical interventions • Brief focused assessment to rule out injury
Teen with Throughand-Through GSW (Arm/Flank) Live actor	Delayed / Intermediate Priority	• Expose wound; obtain baseline vitals • Apply tourniquet if indicated; pack wound if appropriate • Control bleeding; monitor perfusion and mental status • Keep warm and calm
Teen with Leg Amputation + Abdominal Wound Task trainer/live actor mix	Immediate / Critical – Life-Saving Priority	• Apply tourniquet until bleeding stops – use 2nd if needed, time the tourniquet placement • Pack the abdominal wound • Prepare for EMS arrival and reassess VS
Teen with Chest GSW in VFib Arrest High fidelity mannikin	Immediate / Critical – LifeSaving Priority	• Retrieve AED immediately • Initiate high-quality CPR • Apply AED, analyze rhythm, deliver shock if indicated • Coordinate roles, maintain compressor rotation • Prepare for EMS arrival and reassess VS
*Victims: athletic event*		
Hysterical Teen with Emesis Live actor	Minor / Low Priority	• Reassurance, emotional support • Monitor for aspiration risk; lateral positioning as needed • Assign staff member to stay with patient
Teen with Open Tib/Fib Fracture with active brisk bleeding (from student collision) Live actor with moulage	Delayed / Intermediate Priority	• Control bleeding; apply tourniquet if brisk arterial bleeding • Splint fracture using available field materials • Assess distal pulses/sensation before and after splinting
Teen with CHI and Seizure (from student collision) Live actor	Immediate / High Priority	• Seizure safety measures; protect airway • Maintain C-spine precautions due to mechanism • Lateral positioning for emesis • Monitor airway, breathing, color, and perfusion
Adult with Syncope → VFib Arrest (Athletic Event) Low Fidelity mannikin	Immediate / Critical – Life-Saving Priority	• Retrieve AED immediately • Initiate high-quality CPR • Apply AED, analyze rhythm, deliver shock if indicated • Coordinate roles, maintain compressor rotation • Prepare for EMS arrival and reassess VS

Our curriculum had goals and objectives related to individual simulation patient care as well as overall approach to the multi-victim event. For individual patient care, SNs recently had cardiopulmonary resuscitation (CPR) and automated external defibrillator (AED) training and were expected to incorporate those skills. The school shooting simulation involved workshop activities such as splinting, Stop The Bleed©, CPR/AED use, and de-escalation. The athletic event involved workshop activities: splinting, Stop the Bleed©, neurological evaluation for concussion with seizure stabilization/treatment, and syncope with VFib (BLS and AED skills).

To address the overall event, participants were expected to evaluate scene safety and control variables, designate a team leader, call 911 with appropriate dispatch information, and assign specific tasks to other “adults” capable of helping. Part of the debrief was focused on how SNs can identify competent individuals to delegate tasks to by using closed loop communication.

There were many ways to approach the simulation construct, and we chose a combination of live actors, task trainers with low and high-fidelity mannequins. A hybrid patient was simulated with a nurse actor (chest and head) and task trainer torso, the lower half having active hemorrhaging from both an abdominal wound and thigh amputation. MegaCode kid was used for the GSW to the chest for the school shooting and Laerdal SIM Man was used for the adult cardiac arrest at the athletic event. Other live actors, playing victims with bleeding wounds or open fractures had appropriate trauma simulation appliques. All “patients” were appropriately moulaged, had vital signs on laminated cards, and were scattered around the scene. Once the SNs were advised the scene safe, they began the simulation. The high-fidelity mannequins had task trainers with capabilities of pulsatile bleeding, requiring SNs to employ compression and tourniquets to injuries. Further, the acquisition and utilization of the nearest AED was expected. Additionally, many of the patients in the simulations had wounds or injuries needing dressing and splinting. The expectation was that SNs would go in as a team and run the resuscitation treating all patients as if they were real.

A 40-min scenario debrief was conducted using the PEARLS framework. We used a standard 1) reactions phase to ease the emotion (one-two word response), 2) analysis phase, and 3) summary [23]. The IP team contributed and shared their specialty specific experience and expertise. While there was limited time to cover everything, the debrief focused on the initial response, quick patient assessment, how and who to prioritize, expectant management awaiting EMS, using available resources, and delegating others to help.

### Simulation observations

Facilitators were asked to share thoughts from each simulation that they debriefed. Two facilitators were assigned to observe each RN-victim interaction/management and report back during the debrief. This information was to include any safety issues or systems events. The simulations were video recorded in real time from different angles. Given the multi-victim scenario and the volume of sound, the resuscitations were videoed partly in succession. At some points only one victim view with management and communication assessment was possible. Further thematic information was discovered in the debrief with SNs engagement and recollections of their actions as well as assigned facilitator observations. The debriefs were recorded in full to substantiate this information. All videos were saved to a secure drive for the purposes of systems review and reporting to the school system health services.

### SN participants

A total of 90 BPSS registered nurses (RNs) participated in the two training sessions: 53 in April 2023 and 37 in April 2024. All BPSS SNs hold at least a bachelor’s degree in nursing, as required by the State of Massachusetts. Of the 49 participants from 2023 who listed credentials, 20 held master’s degrees, including four nurse practitioners (NPs), one DNP, and two PhDs. Participants in 2024 did not specify credentials as this information was not required for participation. Aside from mandatory CPR/BLS certification, no prior formal simulation training had been provided to BPSS SNs.

## Discussion

### Facilitator perspectives on critical response elements

Following the training, program coordinators synthesized faculty observations from both simulation sessions, drawing on each facilitator’s clinical expertise within their respective disciplines. COMET provided a concise report for BPSS on strengths, weaknesses, and opportunities for improvement. The report focused on areas of emphasis needed for SNs, most specifically the primary survey of a trauma response, communication, and systems issues as described below.

Faculty were instructed to observe for participant adherence to evidence-based practice skills during simulations. Overall, authors felt participants had gaps in approaching an MCI and could improve in four key areas: 1) identifying a team leader to manage the scene, 2) initiating CPR immediately and following AED protocols, 3) reassessment and re-triage, and 4) closed loop communication. Moreover, in the minutes before EMS arrival, SNs must use all resources available to control the scene effectively. Included in that role are delegating tasks, triaging injuries, and preparing the patient for transport for further medical care.

### Airway, breathing, and circulation

Facilitators observed that vital signs were not consistently obtained or reassessed in real time, underscoring opportunities to strengthen rapid patient assessment and prioritization. While CPR was generally initiated appropriately, adherence to established resuscitation protocols varied widely (compression timing, poor recoil). Facilitators emphasized the value of delegating CPR to trained bystanders when available, allowing school nurses to maintain scene oversight and coordinate broader response efforts. Participants were also reminded of the importance of knowing AED locations within their own facilities and, when delegating retrieval, to assign the task to two individuals to ensure accountability and efficiency. Finally, discussions highlighted the difficult but essential concept of triage, recognizing when certain patients may be non-survivable and redirecting efforts toward victims most likely to benefit given the available resources.

### Communication

Facilitators in the 2023 pilot observed limited use of closed-loop communication and inconsistent scene assessment or help-seeking behaviors. By contrast, participants from the inaugural pilot repeating the 2024 session, demonstrated stronger leadership, situational control, and more effective communication. Our findings are in line with other disaster school nurse training studies discussed in this manuscript and provides further evidence that simulation training is an ideal modality to train SNs for MCIs.

Faculty from both years emphasized that clear, structured communication is fundamental to efficient scene management. SNs must rapidly assess the situation, determine the number and acuity of victims, and use available resources to manage competing priorities. A quick scene scan should precede decision-making, allowing nurses to delegate tasks such as applying pressure, providing comfort, or crowd control to bystanders while maintaining overall oversight. Delegating the 911 call is critical to ensure accurate, standardized information is conveyed to dispatch/EMS. Closed-loop communication was reinforced as key to confirming task completion and maintaining situational clarity. SNs were also reminded to anticipate communication barriers, such as cellular “dead zones,” and to establish reliable backup methods within their schools. Finally, facilitators highlighted the importance of communicating directly with students, providing reassurance, clear instructions, and calm leadership, to promote safety and emotional stability during the crisis.

### Systems issues

Facilitators identified several system-level challenges that influenced clinical performance and preparedness. SNs must be prepared to remove clothing as needed to fully assess for hidden injuries and ensure that students with known seizure disorders wear medical alert identification and have individualized care plans in place. In cases of suspected cervical spine injury, adherence to proper spinal precautions was emphasized. A key systems concern was the lack of standardization in emergency equipment bags and available medical supplies across schools. Thus, facilitators encouraged nurses to use available resources creatively and to improvise when standard equipment was lacking.

### Program feedback

**Fig. 1:**
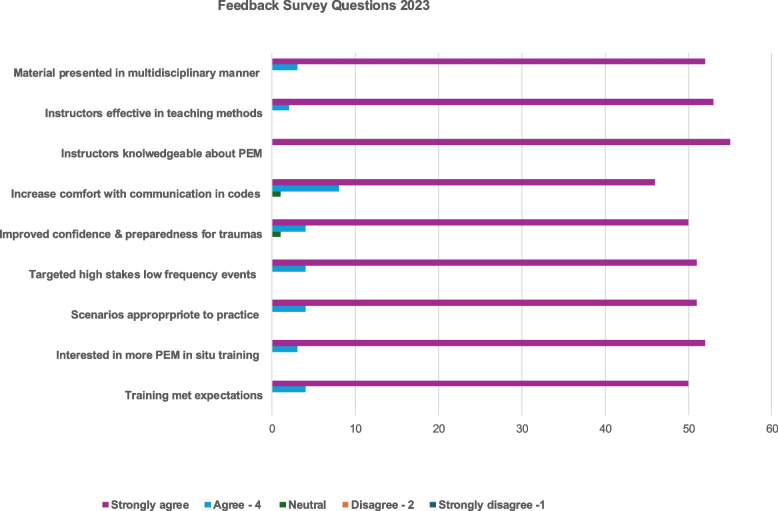
Feedback Survey Questions 2023

**Fig. 2:**
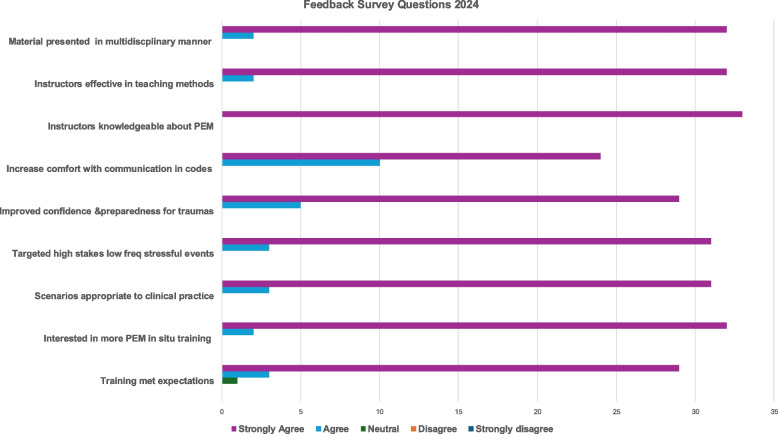
Feedback Survey Questions 2024

Meaningful program improvement relies on participant and facilitator feedback. Survey completion reached 100%, as it was required for continuing education credit. Participants overwhelmingly praised the curriculum and offered constructive suggestions for future iterations. They valued the multimodal learning structure, the involvement of subject matter experts, and the realism of the simulations, which allowed time for questions and deeper discussion. They also provided logistical recommendations to enhance workflow. One comment that resonated, particularly given time constraints, was: “More knowledge and prep for the scenarios, knowing supplies, location of things, walking in with nothing is more daunting,” underscoring the need for additional prebrief time. Figures 1 and 2, display “SNs feedback survey data from 2023 and 2024.” Participants completed ten Likert-scale questions evaluating program logistics and content (legend 1- strongly disagree…5 strongly agree). Across both years, more than 90% of responses were rated 4 or 5, indicating high satisfaction with the sessions. Notably, over 95% of participants reported increased confidence and preparedness for managing emergency events.

Additionally, the team analyzed written feedback from participants and identified six recurrent themes for constructive changes (Table 4). Global suggestions recommended a program run at every BPPS school, at the beginning of the school year, with more simulation time. There was significant interest for more work on de-escalation of critical events. Thematic feedback also revealed increased skill with splinting using every day available resources, newly acquired skills with Stop the Bleed, and the importance of team leader delegation and communication skills for any critical event. One comment that resonated with the authors and something that will be explored in the future is the creation of a School Emergency Response Team (SERT).

**Table 4:** Thematic feedback from RN post session survey questionnaire

Theme	Sample Comments
1. Splinting innovations	• with what we have • never thought of cardboard • using everyday objects • examples of splinting devices • now can swathe • use/recycle things
2. Increased training sessions	• session at all schools • make mandatory • have across districts • should be at the beginning of every school year
3. Clinical approach to trauma	• team leader is essential • reassess patients • meticulous VS • taking charge • role assignment • **create an emergency school response team**
4. Longer session time	• more sim time • more didactic time • love translation of skills to sim but want longer time
5. Stop the bleed knowledge	• pressure and packing were huge • learned tourniquets • got stop the bleed kits and now know what to do • using bleeding mannikins to practice was essential
6. More de-escalation training	• So much mental health issues • Need more dedicated training

### Discussion – Lessons learned

#### Lesson #1

More time for debrief in simulation—there were numerous experts from all healthcare backgrounds and had so much experience and insight to offer. It would be ideal to increase the time spent running the debrief but the maximum session time for the entire program was 4 h. Several comments informed of this, “I do think sessions could be longer – 6 h instead of 4.” Other comments included, “More time and in-depth instructions for simulation…”Wish we had more time to ask questions… great educational experience – not long enough…”.

#### Lesson #2

“Repeat each of the simulations after the debrief”—Repeating each simulation after the debrief aligns with rapid cycle deliberate practice, a technique that uses brief repetitions to build mastery [23]. After the first simulation, BEMS demonstrated their systematic scene approach, including identifying hidden or “well” patients and rapidly determining the number and severity of injured individuals. This modeling provided SNs with a clear expectation for future performance. Ideally, the opening portion of the scenario would be repeated several times to reinforce these behaviors and promote mastery, an approach equally valuable for practicing high-quality CPR within the simulations.

#### Lesson #3

Easy to disseminate—A large proportion of the curriculum was experiential and hands on. There was minimal work for faculty; Stop the Bleed© provides standard slides to certified instructors for use, emergency medicine physicians created visual slides on splinting, child life and social work groups discussed case-based communication techniques, and neurology alike used vignettes to interactively discuss the care appropriate to neurological-based events. The medical actors were recruited from the medical school and friends of EMS. Equipment used to carry out the larger simulations utilized from local simulation programs or borrowed from other medical-based agencies. All of this could be easily replicated with low fidelity simulators especially in lower resource areas.

#### Lesson #4

Several school systems, locally and nationally, have expressed interest in adopting this training but lack the financial resources. Cost remains a significant barrier to replication, and our group is exploring ways to reduce the price point. Nevertheless, school systems must consider investing in emergency preparedness as part of their fiscal planning. As noted, prepared trauma systems achieve superior outcomes through investment in personnel training and protocol development1⁹˒2⁰. Given the increasing frequency of these events, prioritizing such training is essential, and SNs overwhelmingly recommended offering it at least twice per year.

#### Lesson #5

Prior experience builds competency, and repeated simulation exposure supports progression toward mastery [24–26]. In the second year of the curriculum, the number of simulated victims was intentionally increased to create greater complexity and to ensure that all SNs had meaningful, active roles with expanded opportunities to practice key skills. Anecdotally, returning participants demonstrated noticeable growth in leadership, including stronger scene command, more effective delegation, and improved use of closed-loop communication. Given this curriculum had a time constraint of 4 h per session based on school leadership logistics, it would be imperative to repeat the curriculum more often and building upon the foundational skills from the initial event to obtain mastery.

### Limitations

There were several limitations in this curriculum development. This was a small cohort of 90 SNs in a single inner-city system in one state. There were no details on prior formal simulation training outside of BPSS and how that could have impacted the sessions. We know formal MCI training for the SNs was non-existent until our pilot. It would have been preferred for an entire video of each participant-victim interaction, as well as an overall widescreen video of the entire scene. This would allow more specific reviewable data in which we could capture further details. Additionally, the educators were not consistent per session; every facilitator was a volunteer, and it was difficult navigating schedules, thus each half day conducted was a slightly different experience for the SNs. The programming was well received and requested repeatedly, however more sessions with formal evaluative long-term data is needed. Finally, the faculty used their own clinical lens for thematic feedback, not standardized assessment tools, given this was not a formal study. This allows for possible bias from subspeciality perspectives.

### Looking forward

We found this an incredibly valuable opportunity; there needs to be more preparedness in complex medical emergency response if something unforeseen were to occur at school. School systems will inevitably encounter pediatric injuries or medical emergencies for which we know SNs are not fully equipped or prepared. Moreover, during any school-based traumatic event, whether isolated or MCI, the initial medical response will almost always be initiated by SNs. This recognized need, prompted the development of a novel, adaptable curriculum aimed at enhancing readiness for such high-acuity scenarios. Although initially implemented in an urban school district, the curriculum was intentionally designed to be scalable and modifiable, acknowledging that each educational system faces distinct contextual challenges and resource constraints. By sharing the structure and implementation of this multi-modal trauma training model, we aim to provide a replicable framework that can be tailored to other school systems, advancing preparedness, interprofessional collaboration, and the overall safety of children in the school environment.

There are many possible combinations and permutations to each step in creating a similar curriculum, the point being that it can be evolved in so many ways. Any collaborative IP faculty team can modify the session template to the desired timeframe of a particular school system (our group indicated preference for longer sessions). The content of the rotating workshops and simulations can be tailored to the needs of the designated school system. Educators might request a BLS refresher or focus more on mental health concerns, which would be a worthwhile addition to ensure participants are supported to provide support to children during MCIs and themselves during simulation training. Our workshop topics were based on our needs assessment with focus groups. Our inaugural session informed of gaps and weaknesses in our leaners that were targeted in the next iteration of the curriculum. Our simulations focused on school shooting scenarios and athletic event related trauma with medical events intertwined. The scenarios can be more basic or more complex, have a large-scale victim component or much more limited number for ease of execution. Scene environment and goals & objectives can be executed based on context: Ie. war zone, low resource environment etc. The educators can be selected based on session topics. If a behavioral workshop is preferred in the rotating schedule, there can be psychologists and other mental health support. The key point is the curriculum is easily adaptable to meet diverse needs. This innovation in SNs emergency preparedness curriculum is a starting point to support the ability of SNs to care for children before EMS arrives.

This curriculum specifically addresses SNs needs at a campus MCI event. However, we recognize that this content is about bridging care until EMS arrives. In the global community, EMS availability might be sparse or nonexistent in resource limited settings. Furthermore, schools in other parts of the world might not even staff school-based nurses. Thus, this curriculum could be adapted to the country-specific typical emergency response team. Many concepts and workshops could be translated to a course for laypersons as first responders, some of which include: teachers, police, community center workers, or even commercial drivers, all who could potentially be called in for assistance at a local school MCI.

Moving forward, our group plans to survey participating SNs regarding their experiences over the past year, specifically exploring whether the curriculum influenced their response to school-based traumatic events. Future work will focus on a more rigorously designed research project incorporating standardized victims, trained faculty evaluators, and validated assessment tools to objectively measure curricular impact. While initial feedback has been overwhelmingly positive, current evaluation reflects primarily a Level I Kirkpatrick outcome on the Kirkpatrick model [27]. A more comprehensive assessment will be necessary to determine higher-level impacts on performance and system outcomes. Given gaps were identified in leadership and communication the CALM tool could be used to formally measure these skills [28]. Additionally, future simulation iterations could integrate disaster preparedness principles, including formal triage protocols in didactic trainings and incorporated in the simulation experience [29, 30].

Our team is particularly interested in exploring the development of a School Emergency Response Team (SERT) model—an interprofessional framework for coordinated school-based crisis response, analogous to hospital “code teams.” This concept could be further tested through longitudinal training, drills, and tabletop exercises. Finally, future inquiry should address the emotional and psychological effects of such training on SNs, given the increasing prevalence of occupational stress and mental health challenges globally.

## Conclusion

Traumatic, large-scale school events continue to rise in the US, and although less frequent globally, they remain an international concern. With limited on-site medical resources, SNs serve as primary responders during these critical early minutes, making preparedness essential. This curriculum was created to strengthen SNs trauma and medical response skills through a structured, adaptable training model. Early implementation yielded notable improvements in leadership, communication, and scene management, and prompted system-level changes within BPSS, including standardized emergency response kits and provision of *Stop the Bleed©* supplies from BEMS. Growing interest from other school systems further highlights the feasibility and relevance of this approach. The curriculum offers a flexible template that can be tailored to varied school settings, supporting broader dissemination. We hope this model encourages widespread adoption of trauma-readiness training for SNs and contributes to sustained preparedness in the face of evolving school-based emergencies.

## Supplementary Information

Supplementary Material 1.

## Data Availability

Should access be needed for the training day nurse feedback surveys or the final school safety reports (sent to school leadership with specific recommendations), they can be submitted upon request.

## Abbreviations

SNs: School Nurses
BPSS: Boston Public School System
EMS: Emergency Medical Services
BEMS: Boston Emergency Medical Services
US: United States
IP: Interprofessional
CPR: Cardiopulmonary Resuscitation
AED: Automated External Defibrillator
COMET: Community Outreach Mobile Education Training program
GSW: Gunshot Wound
VFib: Ventricular Fibrillation
