# Supplementary material for: School nurse preparedness for critical events – a multi-modal simulation training pilot
Source: Adv Simul (Lond). 2026 Mar 29;11:43. doi: 10.1186/s41077-026-00432-z (PMC13217955; doi:10.1186/s41077-026-00432-z)
Supplement: Supplementary file 1 — Supplementary Material 1. [file 41077_2026_432_MOESM1_ESM.pdf]

COMET

Community Outreach Mobile Education Training

School Nurse Emergency Preparedness for Critical Events

1 UNDERSTAND

Literature confirms an increase in violence, particularly gun violence, in schools across the world (worst in USA). Those in charge of medical care for children at school, mostly school nurses (SNs), are ill-prepared to manage the multi-trauma scenes that ensues following violent acts.

2 SOLUTION

The Boston Public School System (BPSS) sought expert support to provide additions training to their SNs. An interprofessional team identified the problem and weaknesses among BPSS SNs and developed a simulation-based program to provide education for managing these chaotic scenes.

3 EXECUTE

In April 2023, ~60 SNs met in a local school on a Saturday to participate in the training. It was conducted successfully in 2023, and repeated in 2024 for ~30 SNs with additional trauma victims in the simulation

4 OUTCOMES

Primary outcome measures in simulation was adherence to evidence-based practices discussed in workshops such as Stop The Bleed, leader designation, reassessment, emergency services consult, and more. In both years, programming was well received by all participants.

5 FUTURE

In April 2025, participants will be surveyed to determine if the programming impacted their performance as SNs. Methods to reduce cost are ongoing to provide programming to other interested school systems. Long term, authors wish to develop a School Emergency Team to augment outcomes from critical incidents in schools. The present manuscript provides enough templating for other researchers to reproduce.
